# EA Improves the Motor Function in Rats with Spinal Cord Injury by Inhibiting Signal Transduction of Semaphorin3A and Upregulating of the Peripheral Nerve Networks

**DOI:** 10.1155/2020/8859672

**Published:** 2020-11-21

**Authors:** Rong Hu, Haipeng Xu, Yaheng Jiang, Yi Chen, Kelin He, Lei Wu, XiaoMei Shao, Ruijie Ma

**Affiliations:** ^1^Department of Neurobiology and Acupuncture Research, The Third Clinical Medical College, Zhejiang Chinese Medical University, Key Laboratory of Acupuncture and Neurology of Zhejiang Province, Hangzhou, China; ^2^Department of Acupuncture, The Third Affiliated Hospital of Zhejiang Chinese Medical University, Zhejiang Province, Hangzhou, China

## Abstract

Peripheral nerve networks (PNNs) play a vital role in the neural recovery after spinal cord injury (SCI). Electroacupuncture (EA), as an alternative medicine, has been widely used in SCI and was proven to be effective on neural functional recovery. In this study, the interaction between PNNs and semaphrin3A (Sema3A) in the recovery of the motor function after SCI was observed, and the effect of EA on them was evaluated. After the establishment of the SCI animal model, we found that motor neurons in the ventral horn of the injured spinal cord segment decreased, Nissl bodies were blurry, and PNNs and Sema3A as well as its receptor neuropilin1 (NRP1) aggregated around the central tube of the gray matter of the spinal cord. When we knocked down the expression of Sema3A at the damage site, NRP1 also downregulated, importantly, PNNs concentration decreased, and tenascin-R (TN-R) and aggrecan were also reduced, while the Basso-Beattie-Bresnahan (BBB) motor function score dramatically increased. In addition, when conducting EA stimulation on Jiaji (EX-B2) acupoints, the highly upregulated Sema3A and NRP1 were reversed post-SCI, which can lessen the accumulation of PNNs around the central tube of the spinal cord gray matter, and simultaneously promote the recovery of motor function in rats. These results suggest that EA may further affect the plasticity of PNNs by regulating the Sema3A signal and promoting the recovery of the motor function post-SCI.

## 1. Introduction

Spinal cord injury (SCI) and its secondary complications have become a significant social and economic burden on the health care system and patient's families, which mainly leads to irreversible neurological impairment [1]. Although SCI shows some spontaneous recovery of the motor and sensory function, recovery in patients with complete SCI is quite limited and predictable [2]. As an alternative therapy, Electroacupuncture (EA) has been proven to be particularly effective in the rehabilitation of spinal cord injury. According to Dorsher [3], EA can significantly improve the long-term neurological recovery of patients with SCI in the acute stage. Moreover, a previous meta-analysis [4] by our team showed that EA played an active role in the recovery of the neurological function and motor function post-SCI. Additionally, EA on Jiaji (EX-B2) acupoints with the stimulation parameter from 2 Hz/100 Hz has been shown as an antioxidant, anti-inflammation, and antiapoptosis agent, thus promoting axonal regeneration, nerve growth factor improvement, and some gene expressions [5–8]. These effects of EA may have a positive effect on functional recovery post-SCI and can be acknowledged to reduce the risk of secondary spinal cord damage.

Spinal cord injury not only destroys the connectivity of neural circuits but also causes a series of secondary pathological processes, such as inflammation and glial fiber scar, which are chemical barriers to prevent axon regeneration and limit nerve function repair. Nevertheless, the function of the adult spinal cord could be restored by promoting the germination and regeneration of axons and rebuilding the neural circuits [9, 10]. However, the adult central nervous system (CNS) limits the ability of injured neurons to germinate and regenerate [11]. Perineuronal nets (PNNs), a highly structured portion of the extracellular matrix, mostly surround the soma and dendrites of specific type of neurons and play a critical role in protecting nerve growth and regulating neural plasticity [12]. After injury of the CNS, the abnormal accumulation of PNNs can limit the neuroplasticity or axonal regeneration by participating in the formation of glial scar [13, 14]. Therefore, the intervention and regulation of PNNs after SCI may be helpful to the recovery of nerve function and can mediate the spontaneous motor recovery.

Semaphorin3A (Sema3A), as an essential member of the semaphore family, is mainly produced by neurons or astrocytes in the adult CNS, and its polypeptides and neuropeptide receptors are widespread [15]. Recent studies have found that Sema3A is involved in the growth of axons and the formation of new synaptic connections during embryonic development, which is highly aggregated, and migrates to the injured site after SCI [16, 17]. Sema3A can bind to PNNs by chondroitin sulfate-E (CS-E) to prevent the synapses of neurons from crossing the damaged area and prevent the formation of new neuronal connections [18]. Previous studies have shown that EA can alleviate the expression of myelin growth inhibitor and promote nerve regeneration after SCI, and the changes in the connections between these neurons can effectively promote the recovery of the nerve function. So far, our understanding of whether EA can influence neuronal connections and specific mechanisms by interfering with PNN is still very limited.

In order to explore the changes of PNNs mediated by the signaling of Sema3A after SCI, we observed the disintegration of PNNs after the degradation of the Sema3A signal by microinjection of viral vector. Meanwhile, the therapeutic effect of EA was consistent with it.

## 2. Materials and Methods

### 2.1. Reagent and Chemicals

The modified Allen device for a model of spinal cord injury was the Model II-NYU/MASCIS impactor device (W.M.Keck, USA) from the Key Laboratory of Acupuncture and Neurology of Zhejiang Province. The sterilized needles were 0.25 mm × 25 mm from Suzhou Medical Co. Ltd. (JiangSu, China). Acupuncture point nerve stimulator was the HANS-200A from Huawei Co. Ltd. (Beijing, China). Nikon A1R laser scanning confocal microscope was from Nikon Corporation (Nikon, Japan). Frozen microtome was the Thermo NX50 from American Thermo Corporation. The stereotaxic apparatus and isoflurane inhalation were from RWD Life Science Co., Ltd. (Shenzhen, China). The mini-protean vertical electrophoresis and membrane transfer systems were from the U.S. Bio-Rad company. The gel imaging system was Image Quant LAS4000 from the Germany GE Corporation. Microplate Reader was Spectra Max M4 from MeiGu Molecular Co. Ltd.

Sodium chloride, paraformaldehyde, isopropanol, methanol, sucrose, ethanol, penicillin, and Nissl staining solution were from Hanpusi Biotechnology Co., Ltd. (Zhejiang, China). Ponceau S Solution, SDS, Tris, glycine, and ECL Western Blotting Substrate Kit were from Beyotime Science & Technology Co., Ltd. Nembutal and N,N,N′,N′-tetramethyl ethylenediamine (TEMED) were obtained from Sigma-Aldrich (Missouri, USA). The 20 protease inhibitor cocktail tablets were from Roche Diagnostics GmbH (Mannheim, Germany). Pierce ™ BCA Protein Assay Kit was from Thermo Fisher Scientific Inc. (NJ, USA). Difco ™ Skim milk was from Becton, Dickinson, and Company (NJ, USA). The PVDF membrane was from Merck Millipore Ltd. (Billerica, USA). Antibodies to neuropilin1 (WB), HAPLN1, aggrecan, *β-*actin, and Alexa Fluor 488-AffiniPure donkey anti-goat were purchased from Abcam PLC; tenascin-R and neuropilin1 (IF) were from R&D Technology Inc; semaphorin3A was from Genetex Technology Inc; Wisteria floribunda lectin was from Vectorlabs Technology Inc. Anti-rabbit IgG, HRP-linked antibody was from CST, and anti-mouse IgG, HRP-linked antibody was from Jackson. Streptavidin Alexa Fluor Conjugate was from Invitrogen.

### 2.2. Animals

Healthy adult male SD rats (eight weeks old, 200–220 g body weight) were purchased from the Shanghai Xipu Bikai experimental animal Company (animal license No.: SCXK(Shanghai) 2018-0006) and housed in the Laboratory Animal Center of Zhejiang Chinese Medical University accredited by the Association for Assessment and Accreditation of Laboratory Animal Care (AAALAC, animal license No.: SYXK (Zhejiang) 2018-0012). Rats were maintained under controlled conditions with access to food and water ad libitum. All animal experiments were performed in compliance with all relevant ethical regulations for animal testing and research and in accordance with animal protocols approved by the animal ethics committee of Zhejiang Chinese Medical University (ZSLL, 2017-183). All the experimental protocols strictly followed the guidelines of the National Institutes of Health (NIH) on the use of laboratory animals (NIH Publication No. 8023).

### 2.3. SCI Rat Model

To produce a contusive SCI model at T10, rats were placed on their ventral surface in a U-shaped stabilizer, then received a T10 contusion using the MASCIS weight-drop device with a 5 × 10 g/cm gravitational potential energy [19] after a T10 laminectomy. The severity and consistency of the injury were verified by checking the bruise on the spinal cord or tail-flick of rats after weight drop. Rats in the sham group only underwent laminectomy. All animals were injected penicillin (100 U/d) intraperitoneally for 3 days. Then, the rats were returned to clean home cages that were partially placed on a heating pad until they fully recovered from the anesthesia. The manual bladder expression was performed twice daily until the bladder emptying.

### 2.4. Acupuncture Treatment

Rats were submitted to EA treatment at the T9-T11 Jiaji (EX-B2) acupoints [20] which located on the two sides of the spinous process of the dorsal part. All sterilized disposable stainless steel acupuncture needles with a 0.25 mm diameter were inserted as deep as 4-5 mm until the tip of the needle touches the vertebral lamina and then connected with a pair of electrodes from acupuncture point nerve stimulator. The parameters were set as follows: an alternating wave current output (2 Hz/100 Hz), with the intensities remaining at 1 mA that causing slight vibration of the muscles around the treatment areas, was started from the first day after operation, 20 min once daily, for 7 or 14 consecutive days. The rats of the sham group, model group, and AAV group were only bound in the prone position for 20 minutes when the EA group received treatment.

### 2.5. Behavioral Testing

The Basso-Beattie-Bresnahan (BBB) test [21] is judged on a scale of 0-21 (0, complete hind limb paralysis; 21, normal locomotion, Table 1), is based on hind limb movements made in an open field including hind limb joint movement, weight support, plantar stepping, coordination, paw position, and trunk and tail control, and is performed to evaluate the overall basic locomotor performance. Briefly, each rat was placed in an open field and evaluated more than 3 min by two experimenters who were blinded to experimental groups, and one of them counts the total number of scores. Additionally, all rats were assessed before modeling to ensure that there were no baseline defects and averaged into a final score per session.

### 2.6. Sema3A Targets Screening

In order to obtain the effective interference target for Sema3A, the six Sema3A targets of wy2884-2889, the overexpression plasmid wy2890, and the control plasmid wy1720 with the EGFP cDNAs were cloned into plasmid pAAV-MCS. AAV 293cells were cultured in Dulbecco's modified Eagle medium and transport to collect the fraction containing AAV. Then, the target sequences were screened by fluorescence subtracting and Western blot.

### 2.7. AAV Viral Injections

To inhibit the ema3A expression, 0.5 *μ*l AAV2/9-U6-shRNA (Sema3A)-CAG-tdtomato or a negative control AAV2/9-U6-shRNA(luciferase)-CAG-tdtomato virus was injected into the bilateral of the T9 spinal cord using a 10 *μ*l Hamilton syringe, after rats anesthetized with pentobarbital sodium (40 mg/kg, i.p.). The depth of the injection tip was 1.5 mm and keeps in place for another 5 minutes to avoid virus leakage. Vessel and nerve were avoided while injection was done, and virus ultimately titer was 7.5E + 12 v.g./ml.

### 2.8. Nissl Staining

For Nissl staining, 25-*μ*m-thick frozen section was subjected to stain with cresyl violet and dehydrated with different concentrations of ethanol. The number of positive cells in the ventral horn at the epicenter of the lesion 0.5 mm to the injury epicenter was calculated and analyzed by ImageJ software which was averaged into a final score per session.

### 2.9. Western Blotting

Spinal cord tissues of rats were homogenized with radioimmunoprecipitation assay (RIPA) buffer containing proteinase inhibitors, and then the total protein concentration of each sample was determined by using the bicinchoninic acid (BCA) method according to the kit′s instruction. Furthermore, equal amounts of protein from each sample were divorced on 10% SDS-PAGE gels and transferred to polyvinyl difluoride (PVDF) membranes. The membranes were blocked with 5% nonfat milk in TBST containing 0.1% Tween 20 at room temperature for 1 h and then incubated overnight at 4°C with primary antibody: semaphorin3A (1 : 500), neuropilin1 (1 : 1000), HAPLN1 (1 : 1000), aggrecan (1 : 500), Tenascin R (1 : 200), and *β*-actin (1 : 5000). The following day, the membrane was incubated at room temperature for 2 h with the 2 antibodies: anti-rabbit IgG, HRP-linked antibody (1 : 2000) or anti-mouse IgG, HRP-linked antibody (1 : 2000). Finally, the immunoreactivity was detected using enhanced chemiluminescence and visualized with an Image Quant LAS 4000. The density of each band was measured by ImageJ analysis software. The relative expression of the target protein is the target protein (absorbance value)/the internal reference factor of actin (absorbance value), and the results were expressed as mean ± standard deviation.

### 2.10. Immunofluorescence Staining

Transverse spinal cord sections (25 *μ*m) were cut on a frozen microtome, installed on gelatin-coated glass slides as 8 sets of every 5th serial section. Above 25-*μ*m-thick frozen sections were incubated with the following primary antibodies: semaphorin3A (1 : 200), neuropilin1 (1 : 50), and Wisteria floribunda lectin (1 : 100). Signal was detected with the corresponding second antibodies conjugated to Streptavidin Alexa Fluor 555 Conjugate (1 : 200) or Streptavidin, Alexa Fluor 488 conjugate (1 : 200) or Alexa Fluor 488-AffiniPure Goat Anti-rabbit IgG (H + L) (1 : 600) or Alexa Fluor 488-AffiniPure donkey anti-goat IgG (H + L) (1 : 500) and viewed by Nikon A1R laser scanning confocal microscope. In order to quantize the image and keep the uniform microscope setting in the entire image acquisition process, 3-5 images were randomly selected per rat tissue, averaged, and analyzed by ImageJ software.

### 2.11. Statistical Analyses

The statistical significance of the difference between control and experimental groups was determined by one-way ANOVA followed by Tukey Kramer tests which were performed with SPSS.20 (Statistic package for social science) (SPSS Inc., Chicago, USA). Data is shown as mean ± SEM and considered to indicate statistically significant if *P* < 0.05.

## 3. Results

### 3.1. Motor Dysfunction after SCI

**Figure 1:**
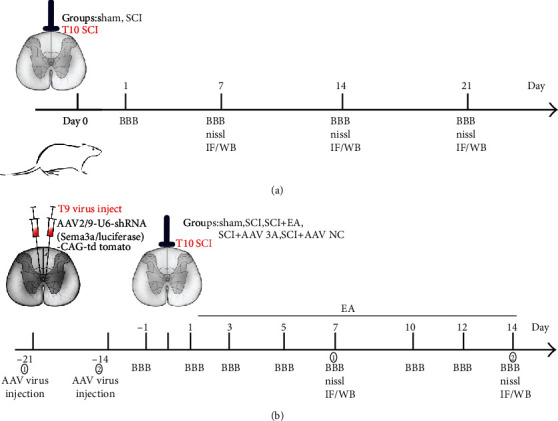
(a, b) Timeline of the experimental protocol.

**Figure 2:**
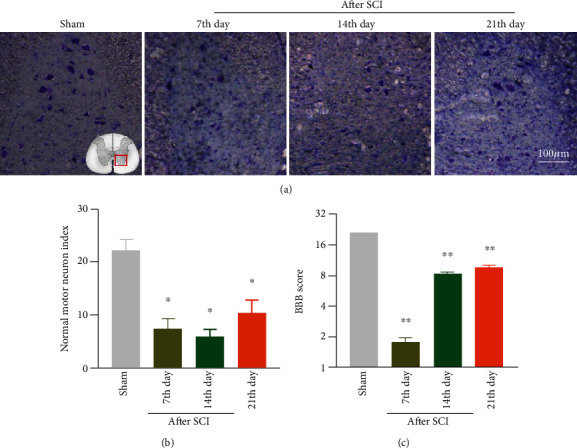
Establishment of the SCI model in rat. After SCI, the motor neurons in the ventral horn of the spinal cord decreased, the Nissl's body became fuzzy, and the motor function decreased. (a) The Nissl staining of the ventral horn of the spinal cord in the sham group and the SCI group represents the figure. (b) The number of surviving motoneurons in the ventral horn of the spinal cord was quantified, *n* = 3. (c) BBB function scores of rats in the sham group and the SCI group, *n* = 10, compared with the sham group, ^∗^*P* < 0.05, ^∗∗^*P* < 0.01. All data are presented as the mean ± SEM.

Firstly, we established the modified rat model of SCI via Allen's method described previously. As shown in Figure 1(a), the BBB score was used to observe the motor function of hind limbs on the 7th, 14th, and 21st days after SCI in rats, and we found the hind limbs of rats completely paralyzed following SCI, while its performance was improved gradually from the second post-SCI week. Specifically, the lower limb motor function was normal with the score of 21 points in the sham group. Unfortunately, the BBB score was among 0-2 points on the 7th day post-SCI which had severe motor dysfunction, and the motor function of hind limbs began to gradually recover after 14 days (Figure 2(c)). Therefore, it can be predicted that the motor function of the hind limbs was obviously impaired after SCI, whereas it can be slightly recovered on account of the limited self-healing ability.

### 3.2. Morphological Changes of the Spinal Cord after SCI

We carried out Nissl staining to evaluate the changes of neuron morphology in the sham group and on the 7th, 14th, and 21st days after SCI. The results showed, compared with the sham group, that Nissl-positive motor neuron number in the ventral horn of the spinal cord after SCI was significantly reduced, and Nissl bodies became fuzzy and gradually recovered on the 21st day after SCI (Figures 2(a) and 2(b)).

### 3.3. The Expression of Sema3A and NRP1 after SCI

**Figure 3:**
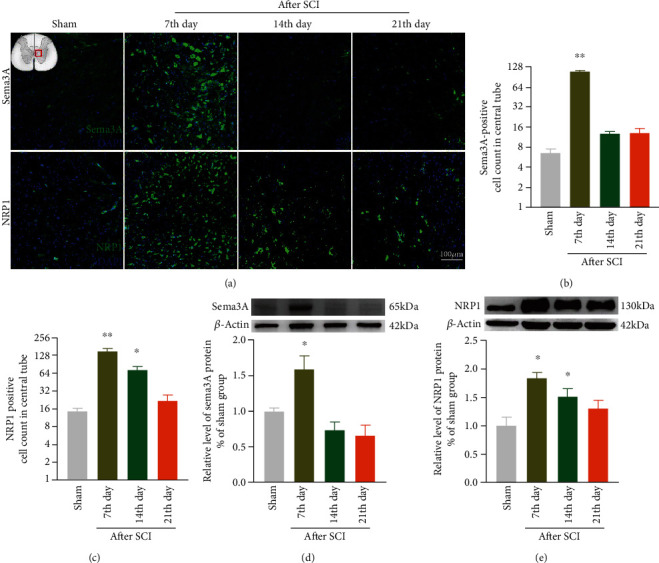
The expression of Sema3A and its receptor NRP1 were upregulated around the central gray matter of the spinal cord after SCI. (a) The results of immunofluorescence showed the expression of Sema3A and its receptor NRP1 around the central canal of the spinal cord gray matter in the sham group and the SCI group. (b, c) Quantification of immunofluorescence data in panel (a), *n* = 3. (d, e) Representative bands and statistics of Sema3A and NRP1 in the spinal cord by Western blotting, *n* = 5, compared with the sham group, ^∗^*P* < 0.05, ^∗∗^*P* < 0.01. All data are presented as the mean ± SEM.

To quantify the expression of the Sema3A signal in the injury site of spinal cord, Sema3A and NRP1 immunotherapy were performed. Subsequently, our immunofluorescence study showed that the Sema3A signal around the central canal of the spinal cord gray matter increased abnormally compared with the sham group on the 7th day post-SCI, but there was no significant differences on the 14th and 21st day after SCI. NRP1 also significantly increased around the central canal on the 7th day post-SCI, and it was downregulated on the 14th day after SCI while still higher than that of the sham group; however, no significant difference was observed on the 21st day after SCI compared with the 14st day in the post-SCI group (Figures 3(a) and 3(b)). We further examined the expression of Sema3A and NRP1 in the SCI rats by Western blotting, and the results were consistent with that of immunofluorescence (Figures 3(c) and 3(d)).

### 3.4. The Expression of PNNs after SCI

**Figure 4:**
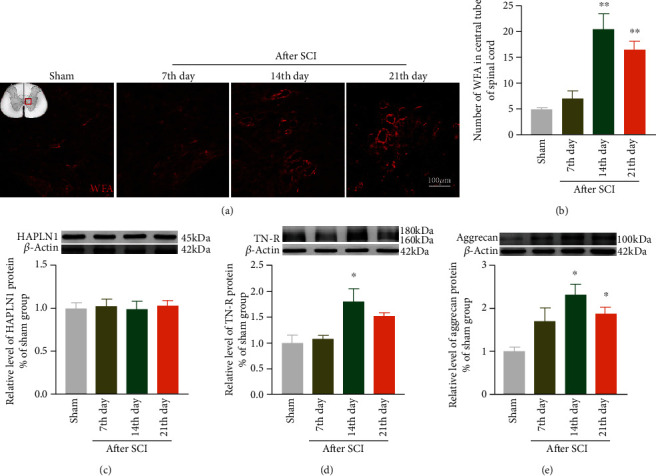
Expression changes of PNNs and their related proteins TN-R, HAPLN1, and aggrecan in the spinal cord after SCI. (a) Immunofluorescence representative diagram of changes in expression levels of WFA around the central tube of the spinal cord in the SCI group and sham group. (b) Quantification of the immunofluorescence data in panel (a), *n* = 3. (c) Representative bands and statistics of TN-R, HAPLN1, and aggrecan in the spinal cord by Western blotting, compared with the sham group, ^∗^*P* < 0.05, ^∗∗^*P* < 0.01. All data are presented as the mean ± SEM.

It has been demonstrated that the aggregation of PNNs wrapping around soma and dendrites after the CNS injury hinders the neuronal axon regeneration and limits the nerve plasticity. We labeled PNNs in the spinal cord by wisteria floribunda agglutinin (WFA) which can be bind to *N*-acetylgalactosamine (GalNAc) in most PNN polysaccharide chains. Importantly, immunofluorescence results were similar to previously reported which showed that WFA was upregulated around the central canal of the spinal cord gray matter post-SCI and reached the peak on the 14th day (Figures 4(a) and 4(b)). In addition, we examined the expression of the main structural proteins in PNNs, including TN-R, HAPLN1, and aggrecan by Western blotting. We found HAPLN1 was not significantly changed after SCI, while TN-R and aggrecan were significantly upregulated on the 14th day after SCI compared to the sham group (Figures 4(c)–4(e)).

### 3.5. Sema3A Targets Screenings

**Figure 5:**
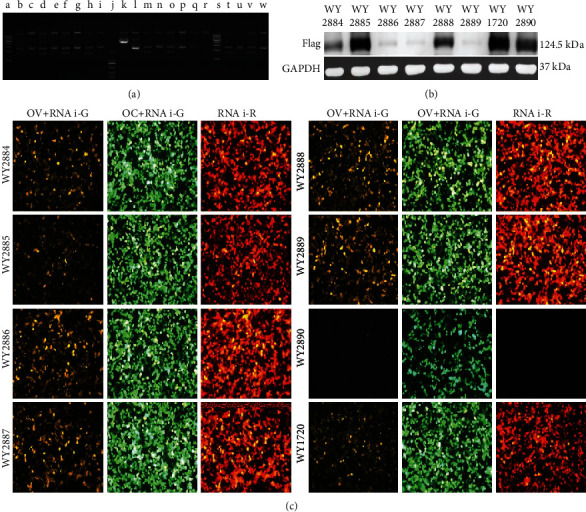
Sema3A in vitro target screening. (a) wy2884-2889 plasmid enzymatic digestion validation, using the enzymatic digestion site: Smal, (a and s: 1Kb DNA ladder, the bands from bottom to top are 1Kb, 2Kb, 3Kb, 4Kb, 5Kb, 6Kb, 7Kb, 8Kb, 9Kb, and 10Kb; J: DL 2000 DNA marker, the bands from bottom to top are 100 bp, 250 bp, 500 bp, 750 bp, 1Kb, and 2Kb; b, d, f, h, k, m, o, q, t, and v were, respectively, the bands after wy2884-2889 plasmid digestion,and a, c, e, g, i, l, n, p, r, u, and w were the control bands of wy2884-2889 plasmid without enzyme digestion, and the size of each band was in line with the expectation). (b) The interference group sample wy2884-2889, the interference control samples WY1720, and the overexpressed samples WY2890 Western blotting bands. The Flag signal was obvious, and the size was 124.5 kDa. As a whole, the wy2887 group had the least protein and the best interference effect. The WY2886 and WY2889 groups also had less protein. (c) Representative diagram of the interference group wy2884-2889, the interference control sample WY1720 ,and the overexpressed sample WY2890 were cotransfected into 293 T cells in vitro of fluorescence attenuation detection. The results showed that the negative control wy1720 of the interfering target had no knockdown ability. The target sequences of WY2886, WY2887, and WY2889 in the experimental group wy2884-2889 had a strong knockout ability.(OV: wy2890: pAAV-CMV_bGI-Sema3A-EGFP-3Flag-WPRE-hGHpA; OC: wx963: pAAV-CMV_bGI-EGFP-3Flag-WPRE-hGHpA).

**Figure 6:**
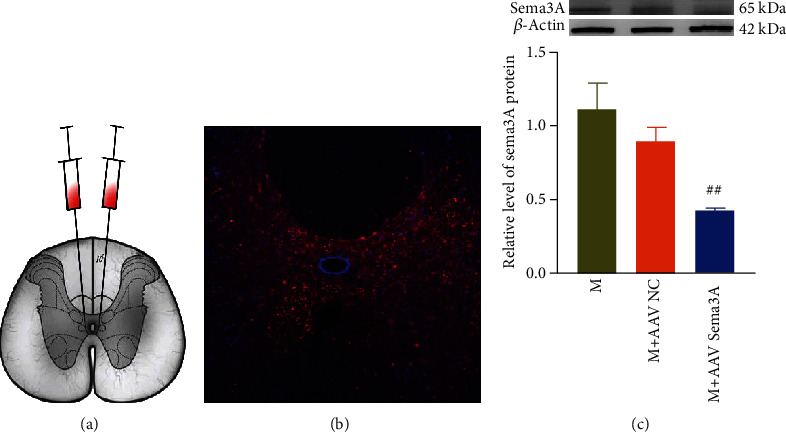
The expression of Sema3A around the central tube of the spinal cord is decreased after knockdown Sema3A. (a) Schematic diagram of virus injection. (b) The microscopic representative diagram of the T10 expression at 4 weeks after virus injection. (c) The quantitative diagram represents bands of Sema3A Western blotting in the T10 spinal cord at 4 weeks after virus injection, *n* = 3, compared with the SCI group, ##*P* < 0.01. All data are presented as the mean ± SEM.

To further verify the impact of Sema3A post-SCI, we designed 6 sets of SNCA-shRNA sequence plasmids with GFP fluorescent tags and integrated them into an adeno-associated virus (AAV), then transfected them into HEK293 cells to observe the degree of translated. We utilized fluorescence attenuation detection cotransfect 293 T cells in vitro with the interference group, the interference control sample wy2884-2889, the interference control sample WY1720, and the overexpressed sample WY2890. It was found that the negative control WY1720 of the interference target had no ability to knockdown, while the target sequences of WY2886, WY2887, and WY2889 in the experimental group had a strong knockdown ability (Figure 5(c)). Western blotting was further used to check the efficiency of each target, and the result displayed WY2887 had the least amount of protein as well as the best interference effect (Figure 5(b)). Therefore, we considered wy2887 is the most suitable one among the six sequences of wy2884-2889 (Table 2). Hence, we chose wy2887 to package AAV2/9-U6-shRNA (Sema3A)-CAG-tdtomato for the following experiment. To verify the knockdown efficiency of Sema3A shRNA in rats, AAV2/9-U6-shRNA(Sema3A)-CAG-tdtomato was injected into the T9 spinal cord by stereoscopic microinjection for 21 days before modeling (Figure 6(a) and 6(b)), AAV2/9-U6-shRNA(luciferase)-CAG-tdtomato was used as a comparison, and samples were extracted on the 7th day after SCI for Western blotting. It was displayed that AAV2/9-U6-shRNA (Sema3A)-CAG-tdtomato could significantly decrease the expression of Sema3A after SCI (Figure 6(c)).

### 3.6. Inhibition of Sema3A Promotes Functional Recovery after SCI by Reducing the Accumulation of PNNs at the Injury Site

**Figure 7:**
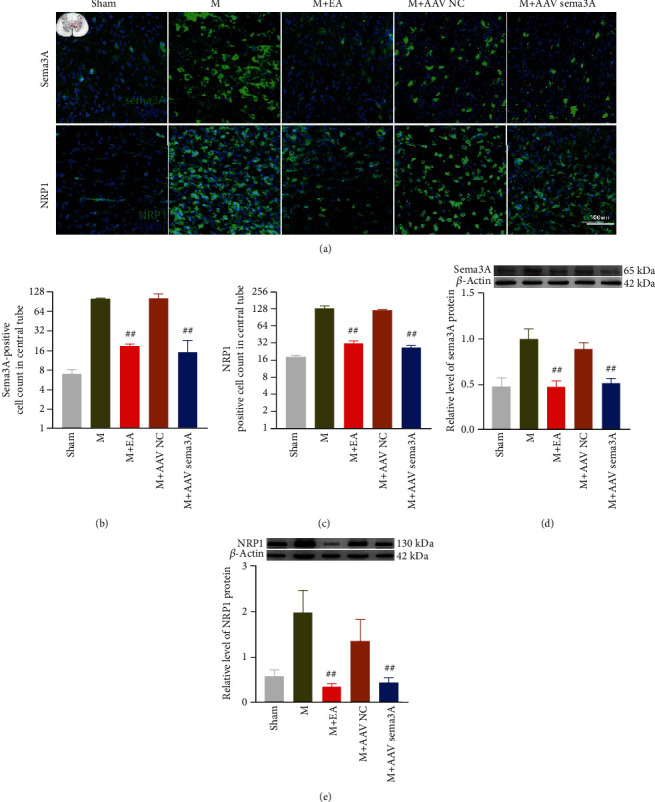
EA and Sema3A knockdown downregulated the expression of Sema3A and its receptor NRP1 around the central canal of the spinal cord. (a) A representative diagram of Sema3A and its receptor NRP1 expression around the central tube of gray matter in the spinal cord in the sham group, M + EA group, M + AAV Sema3A group, and M + AAV NC group was shown by immunofluorescence. (b, c) Quantification of the immunofluorescence data in panel (a), *n* = 3. (d, e) The representative band and statistic of Sema3A and NRP1 in the spinal cord, *n* = 5, compared with the SCI group, ##*P* < 0.01. All data are presented as the mean ± SEM.

**Figure 8:**
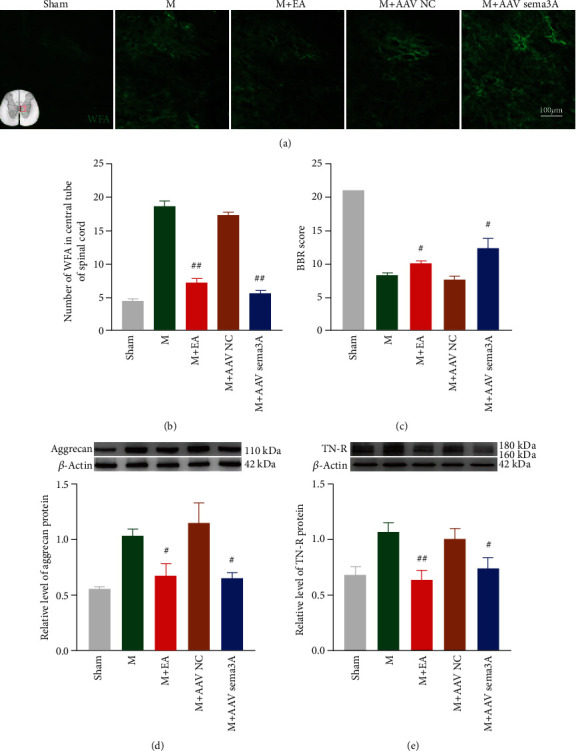
The expressions of WFA and TN-R and aggrecan in the spinal cord were downregulated by EA and Sema3A knocking. (a) A representative chart of the changes of the WFA expression around the central tube of the spinal cord in the sham group, 14 days after SCI, M + EA group, M + AAV Sema3A group, and M + AAV NC group, was shown by immunofluorescence. (b) Quantification of the immunofluorescence data in panel (a), *n* = 3. (c) The motor function score of each group at day 14. (d, e) The representative band and statistics of aggrecan and TN-R in the spinal cord, *n* = 5, compared with the M group, #*P* < 0.05, ##*P* < 0.01. All data are presented as the mean ± SEM.

To clarify the role of Sema3A, we injected AAV2/9-U6-shRNA (Sema3A)-CAG-tdtomato into T9 spinal cord rats 21 days or 14 days before modeling according to the experimental plan to knock down the expression of Sema3A (Figure 1(b)). AAV2/9-U6-shRNA(luciferase)-CAG-tdtomato was used as negative control, then observed the changes of PNNs around the central canal of the spinal cord at the injured area and the motor function of the hindlimbs of the rats. Immunofluorescence and Western blotting both recovered that knockdown Sema3A could significantly downregulate the expression of Sema3A and its receptor NRP1 (Figure 7). Indeed, WFA around the central canal was downregulated after knocking down Sema3A (Figures 8(a) and 8(b)), and the expressions of TN-R and aggrecan in the SCI area were also deregulated, but the control virus group had no significant effect (Figures 8(d) and 8(e)).

**Figure 9:**
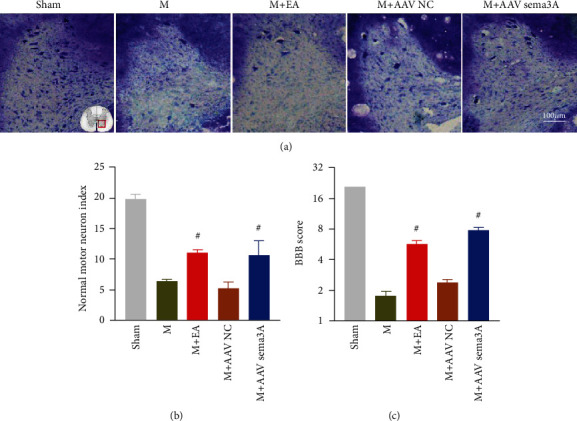
EA or Sema3A knockdown can promote the functional recovery after SCI in rats. (a) The figure represents the Nissl staining of the ventral horn of the spinal cord (b) The number of surviving motoneurons in the ventral horn of the spinal cord was quantified by Nissl staining, compared with the SCI group, #*P* < 0.05, *n* = 3. (c) The motor function score of each group at day 7, compared with the SCI group, #*P* < 0.05, *n* = 10. All data are presented as the mean ± SEM.

Morphologically, the Nissl staining result was reminded that the number of Nissl-positive motor neurons in the ventral horn of the spinal cord about the group of knocking down Sema3A was notably increased compared with the SCI group (Figures 9(a) and 9(b)). Functionally, we found that the BBB score was significantly higher than the SCI group when we deliberately downregulated the expression of Sema3A in the injured spinal cord, except for the control virus group, which suggested that downregulation of Sema3A could promote the recovery of the lower limb motor function in the SCI rats (Figures 8(c) and 9(c)).

### 3.7. EA Treatment Promotes Functional Recovery after SCI, Possibly by Regulating Sema3A to Reduce the Accumulation of PNNs

To evaluate whether EA could meliorate the abnormal aggregation of PNNs and Sema3A, we used immunofluorescence and Western blotting to detect the influence of EA treated on SCI. Excitingly, the changes of molecular biology in the injured spinal cord of SCI rats after EA treatment were similar to that of knockdown Sema3A. Immunofluorescence showed that the aggregation of Nrp1, Sema3A, and WFA around the central canal of the spinal cord gray matter in the EA-treated group was lower than that in the SCI-treated group (Figures 7(a)–7(c) and 8(a) and 8(b)). Furthermore, these results were verified by Western blotting which wonderfully demonstrated that the protein expression of Sema3A, Nrp1, TN-R, and aggrecan in the spinal cord of the EA-treated group was evidently less than the SCI group (Figures 7(d) and 7(e) and 8(d) and 8(e)).

In addition, damaged motor neurons in the ventral horn of the injured spinal cord were properly repaired after EA treatment. Specifically, as far as histomorphology is concerned that the number of Nissl-positive motoneurons in the ventral horn of the spinal cord, compared to the SCI group, was significantly augmented by EA treatment (Figures 9(a) and 9(b)). In terms of the motor function, EA has the same effect as those who were knocked down the expression of Sema3A in the injury spinal cord, which can actively improve the motor function of hind limbs in rats with SCI. Although the BBB score of the EA group was slightly lower than the Sema3A knockdown group, it was still visibly greater than the SCI group, especially after the 5th day under intervened (Figures 8(c) and 9(c)).

### 3.8. Discussion

PNNs and Sema3A are widely known for their capacity to limit nerve plasticity after CNS injury. In this study, we found that motor neurons in the ventral horn of the injured spinal cord segment decreased, and PNNs and Sema3A as well as its receptor NRP1 aggregated around the central tube of the gray matter of the spinal cord. When the expression of Sema3A at the damage site was knocked down, NRP1's expression was also downregulated, accompanied by the PNNs concentration decreased. The expression of TN-R and aggrecan was also reduced; meanwhile, the BBB motor function score dramatically increased. In addition, Electroacupuncture can reverse the high upregulation of Sema3A and NRP1 after spinal cord injury, reduce the accumulation of PNNs around the central canal of gray matter, and promote the recovery of the motor function.

It is worth noting that PNNs, as the main external environment of the central nervous system, are mainly composed of hyaluronic acid (HA), proteoglycan 1 (hapln1), tenascin-r (TN-R), and chondroitin sulfate proteoglycan (CSPG) [22]. They are activity dependent and form around the soma and proximal neurites presenting at the closure of critical periods during development and involve many homeostasis functions, for example, neuroprotection. Biochemical analysis suggests that PNNs are present in 30% of ventral horn motor neurons in the spinal cord [23–25]. TN-R as a member of the tenascin family in the CNS is closely related to the development and plasticity of nervous and the migration of nerve cells. Aggrecan is one type of CSPGs. It can be connected with the lectin domain of CSPGs to form an organized PNN backbone, which is crucial for the structure of PNNs [26, 27]. Studies have shown [28, 29] that in SCI models, the expression of CSPGs is upregulated and gradually migrated to the injury site, interacting with astrocytes, microglia, and macrophages to form a glial scar and inhibit axonal regeneration. In addition, inhibition of the expression of TN-R and aggrecan is conducive to the reconstruction of synapses between neurons and the repair of the spinal cord function after SCI [30, 31].

Interestingly, PNNs constitute a physical barrier between neurons and extracellular cells which impede nerve repair in the model of SCI, while enzymatic removal of PNNs can promote functional recovery [32]. Massey and coworkers [33, 34] found that PNNs were obviously upregulated around dorsal column nuclei neurons of rats following a mid-cervical dorsal column tract lesion postinjury, while these upregulated PNNs restricted the plasticity and allowed sprouting into its denervated portions from the intact sensory axons with their degrade via injection of ChABC which can acutely remove CS-GAGs. So, changes in the PNN may be beneficial to regulating the remodeling and plasticity that occur in SCI for the animal to acquire some degree of locomotor functional recovery.

Recent finding [35] indicates that Sema3A, as an inhibitor of axon connection, contains receptor neuropilin1 (NRP1) which is the extracellular receptor and expressed on the dendrites and axons of neurons. It is rarely expressed in normal conditions, but significantly increased after SCI, leading to the collapse of nerve growth cone and inhibiting the connection between neurons only under the action of NRP1 [36, 37]. Studies on the relationship between Sema3A and PNNs have shown that [38–40] Sema3A may possible be a potentially part of PNNs in regulating neuronal plasticity after SCI, which plays a key role in nerve remodeling by highly affinity binding to PNNs through chondroitin sulfate E.

In this experiment, we first evaluated the changes of the Sema3A signal and PNNs in SCI rats and further studied the potential relationship between them and the effect of EA. We found that the number of motor neurons decreased and motor dysfunction after SCI, accompanied by significantly increases in the expressions of PNNs, TN-R and aggrecan, Sema3A, and NRP1, but no significant changes in HAPLN1, compared to the sham group. Subsequently, in order to further study the correlation between the Sema3A signal and PNNs in the rat spinal cord tissue after SCI, as well as the effect of EA intervention, we packaged the Sema3A target sequence selected in vitro and injected it into the injured site to knock down the expression of Sema3A as a positive control for the EA intervention. We found that when rats passively reduce the expression of Sema3A around the central canal of gray matter or receive EA at Jiaji points, the Sema3A signal, WFA, TN-R, and aggrecan aggregation in the spinal cord tissue are significantly reduced and with the recovery of the motor function of both lower limbs.

EA as a complementary method to treat SCI has been widely used and is known for its benefits in synapse formation, neural rehabilitation, and restoration, which may be attributed to its enhancement of neurotrophic factor secretion, antioxidation, anti-inflammation, and antiapoptosis [8, 41, 42]. The frequency and wave type of EA are of great importance for functional recovery of patients with SCI. EA with loose-dense wave can significantly promote nerve regeneration and repair of SCI rats, speed up the removal of free radicals, enhance blood circulation, and reduce the secondary injury of SCI to promote the recovery of the motor function significantly [43]. Importantly, there is increasing evidence that [44] 2 Hz/100 Hz loose-dense wave EA plays a positive role in motor functional recovery of SCI which can promote nerve regeneration and repair.

In the theory of traditional Chinese medicine, Jiaji points are located between the governor vessel (GV) and the bladder meridian of foot-taiyang (BL). In the human body, there are 34 acupoints belong to huatuo Jiaji points which locate on 0.5 inch lateral from the first thoracic vertebra to the fifth lumbar vertebra. Therefore, needling insert into Jiaji points can not only regulate the qi of the GV and the BL but also adjust the balance of qi and blood in the zang-fu organs, so as to dredge and smooth the passage of the meridian. From the anatomy structure, EA on Jiaji points can stimulate the corresponding posterior ramus of the spinal nerve arising from the lower vertebrae. Our previous results also confirmed that 2 Hz/100 Hz EA stimulation of Jiaji points can reduce the expression of myelin growth inhibitor and effectively promote the regeneration of nerve and axon after SCI [45, 46]. Despite the fact that some previous studies have confirmed the role of EA in promoting functional recovery after SCI, the mechanism of whether EA can promote neurological recovery by interfering with PNN changes after SCI is still in the exploratory stage.

In this study, we found the expression levels of aggrecan and TNR protein in PNNs were dramatically reduced, as well as Sema3A and NRP1, after 2 Hz/100 Hz alternating wave acupuncture treatment, which was consistent with the trend after the knocking down of Sema3A in the damaged site. Taken together, the data of our study have showed that regulating the expression of Sema3A after SCI can affect the plasticity of PNN and is beneficial to the repair of the motor function, and Jiaji EA may regulate PNN plasticity through Sema3A and improve the locomotor function of the paralyzed hind limbs finally.

## 4. Conclusions

In summary, the results showed that EA at Jiaji points could improve the recovery of the motor function after SCI and has a positive therapeutic effect on SCI. EA therapy for SCI may affect the plasticity of PNNs by regulating the Sema3A signal transduction. This study ultimately reveals a new sight for the intervention effect of EA on spinal cord injury; however, the precise mechanism of the negative correlation between PNNs and motor function recovery post-SCI remains to be further explored.

## Figures and Tables

**Table 1:** Basso-Beattie-Bresnahan locomotor rating score.

Score	The ability of the lower limb motor
0	There is no visible hindlimb (HL) movement
1	Light movement of one or both joints, usually hip and/or knee
2	Broad movement of one joint or joint and slight movement of the other
3	Extensive movement of the two joints
4	Light movement of three joints
5	Light movement of two joints and wide movement of the third
6	Broad movement of the two joints and light movement of the third
7	The extensive movement of all three joints of HL
8	The ball of the foot without weight support or without weight support
9	The soles of the feet occasionally bear the weight of the ground (for example, when stationary), frequent or consistent load-bearing movements of the dorsal claw, without the soles of the feet supporting the movement
10	Paw surface occasionally moves with load bearing without FL-HL coordination
11	Paw surface has more load-bearing movement and no FL-HL coordination
12	More load-bearing movement and occasional FL-HL coordination on the paw surface
13	Common paw-bearing movement and frequent FL-HL coordination
14	Continuous palm-surface-bearing movement with consistent FL-HL coordination, or common palm-surface movement, continuous fore-hind limb coordination, and occasionally dorsal claw movement
15	Continuous paw and palm-bearing movement and consistent FL-HL coordination, no or occasional ground grasping movement in the forward motion of the forelimbs, and the position of the main claw parallel to the body at the initial contact
16	In the gait, the continuous paw landing and the coordinated movement of the front and rear limbs are common in the process of grasping the ground; the main claw position is parallel to the body at initial contact and rotates after load transfer
17	In the gait, the continuous paw landing and the coordinated movement of the front and rear limbs are common in the process of grasping the ground; the main claw position is parallel to the body at initial contact and load transfer
18	In the gait, the continuous paw touches the ground in a coordinated manner with the front and rear limbs. In the process of progress, the continuous paw grasps the ground. The position of the main paw is parallel to the body at the initial contact
19	In the gait, the continuous paw touches the ground in a coordinated manner with the front and rear limbs. The continuous paw grasps the ground in the process of advancing. The position of the main paw is parallel to the body at the initial contact and load transfer
20	The position of the main claw is parallel to the body during initial contact and weight transfer. The trunk is unstable, and the tail kept cocking up
21	The position of the main claw is parallel to the body at the initial contact and load transfer, and the trunk is stable and the tail kept cocking up

**Table 2:** Sema3A target screening sequence.

No.	Target sequence	Plasmid
1	GGAAAGAACAATGTGCCAA	WY2884
2	CCATCCAATTTGCACCTAT	WY2885
3	CCTGAAGATGACAAAGTAT	WY2886
4	GCTAGAATAGGTCAGATAT	WY2887
5	GCAATGGAGCTTTCTACTA	WY2888
6	GGATGAGTTCTGTGAACAA	WY2889

## Data Availability

All the data used to support the findings of this study are included within the article.

## Additional Points

*Author statements.* The manuscript is original, has not been submitted to or is not under consideration by another publication, has not been previously published in any language or any form, including electronic, and contains no disclosure of confidential information or authorship/patent application/funding source disputations.
